# Poly[[diaqua­tris(μ_2_-4,4′-bi­pyridine)­bis[μ_2_-2-(carb­oxylato­methyl­sulfan­yl)nicotinato]dicobalt(II)] 1.3-hydrate]

**DOI:** 10.1107/S1600536813015262

**Published:** 2013-06-08

**Authors:** Rui-Qin Li, Xiao-Juan Wang, Yun-Long Feng

**Affiliations:** a Zhejiang Key Laboratory for Reactive Chemistry on Solid Surfaces, College of Chemistry and Life Science, Zhejiang Normal University, Jinhua, Zhejiang 321004, People’s Republic of China

## Abstract

The title complex, [Co_2_(C_8_H_5_NO_4_S)_2_(C_10_H_8_N_2_)_3_(H_2_O)_2_]·1.3H_2_O, was synthesized under hydro­thermal conditions. The Co^II^ ion is six-coordinated in a slightly distorted octa­hedral environment resulting from two carboxyl­ate O atoms of two 2-carb­oxy­methyl­sulfanyl nicotinate (2-CMSN^2−^) anions, one water mol­ecule and three N atoms of three 4,4′-bi­pyridine ligands, with one 4,4′-bi­pyridine ligand situated on a centre of inversion. Two neighboring Co^II^ ions are linked by two anions, giving a dinuclear [Co_2_(2-CMSN)_2_] subunit with a Co⋯Co separation of 6.8600 (3) Å. The dinuclear subunits are joined by bridging 4,4′-bi­pyridine linkers, generating a three-dimensional network structure. Disordered water mol­ecules are situated in the free space of this network. O—H⋯O hydrogen bonding within and between the subunits enhances the stability of the structure.

## Related literature

For general background to coordination polymers, see: Wang *et al.* (2004 ▶). For crystal structures of related compounds based on 2-mercaptonicotinic acid, see: Sun *et al.* (2011 ▶). For complexes derived from the 2-H_2_CMSN ligand, see: Jiang *et al.* (2010 ▶, 2012 ▶).
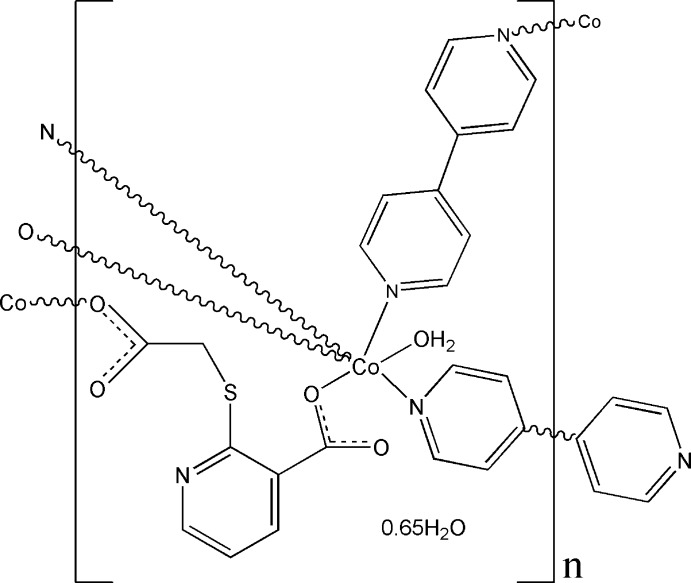


## Experimental

### 

#### Crystal data

[Co_2_(C_8_H_5_NO_4_S)_2_(C_10_H_8_N_2_)_3_(H_2_O)_2_]·1.3H_2_O
*M* *_r_* = 534.13
Monoclinic, 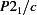

*a* = 10.2211 (1) Å
*b* = 17.1355 (2) Å
*c* = 16.4142 (2) Å
β = 125.484 (1)°
*V* = 2340.92 (5) Å^3^
*Z* = 4
Mo *K*α radiation
μ = 0.87 mm^−1^
*T* = 296 K
0.34 × 0.20 × 0.11 mm

#### Data collection

Bruker APEXII CCD diffractometer
Absorption correction: multi-scan (*SADABS*; Bruker, 2006 ▶) *T*_min_ = 0.814, *T*_max_ = 0.912
38093 measured reflections
5439 independent reflections
4772 reflections with *I* > 2σ(*I*)
*R*_int_ = 0.025

#### Refinement

*R*[*F* ^2^ > 2σ(*F* ^2^)] = 0.033
*wR*(*F* ^2^) = 0.093
*S* = 1.05
5439 reflections
315 parameters
3 restraints
H-atom parameters constrained
Δρ_max_ = 0.68 e Å^−3^
Δρ_min_ = −0.53 e Å^−3^

### 

Data collection: *APEX2* (Bruker, 2006 ▶); cell refinement: *SAINT* (Bruker, 2006 ▶); data reduction: *SAINT*; program(s) used to solve structure: *SHELXS97* (Sheldrick, 2008 ▶); program(s) used to refine structure: *SHELXL97* (Sheldrick, 2008 ▶); molecular graphics: *DIAMOND* (Brandenburg, 2008 ▶); software used to prepare material for publication: *SHELXTL* (Sheldrick, 2008 ▶).

## Supplementary Material

Crystal structure: contains datablock(s) I. DOI: 10.1107/S1600536813015262/wm2744sup1.cif

Structure factors: contains datablock(s) I. DOI: 10.1107/S1600536813015262/wm2744Isup2.hkl

Additional supplementary materials:  crystallographic information; 3D view; checkCIF report

## Figures and Tables

**Table 1:** Hydrogen-bond geometry (Å, °)

*D*—H⋯*A*	*D*—H	H⋯*A*	*D*⋯*A*	*D*—H⋯*A*
O1*W*—H1*WA*⋯O3^i^	0.85	1.89	2.663 (2)	150
O1*W*—H1*WB*⋯O2	0.85	1.90	2.682 (2)	152

Symmetry code: (i) 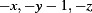.

**Fig. 1.**
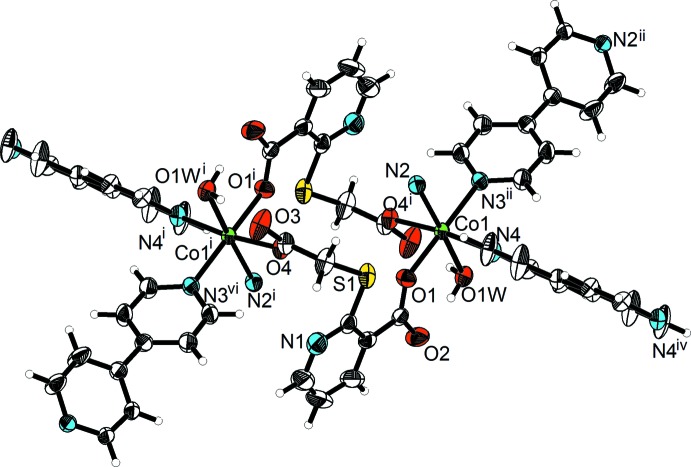
The coordination environment of the Co2+ ion in the title compound and the bridging character of the ligand. Displacement ellipsoids are drawn at the 30% probability level. [Symmetry codes: (i)-x, -y - 1, -z; (ii) x + 1, -y - 1/2, z + 1/2; (iv) -x, -y - 1, -z + 1; (vi) -x-1, y-1/2, -z-1/2.]

**Fig. 2.**
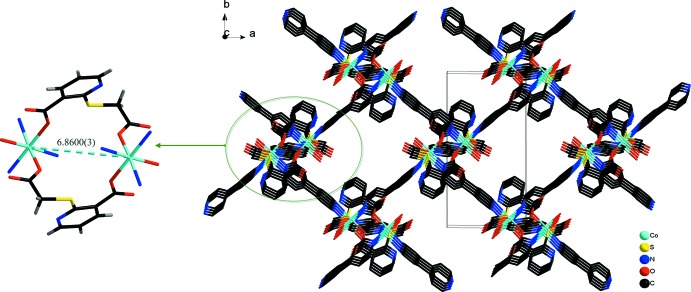
The dinuclear [Co2(2-CMSN)2] subunit (left), and the three-dimensional network of the title compound (right) viewed approximately down [001].

## supplementary crystallographic information

### Comment

The construction of coordination polymers has aroused attention due to their potential applications, fascinating topologies and entanglement motifs (Wang *et al.*, 2004).

2-Mercaptonanicotinic acid (2-H_2_MN) is a multifunctional ligand containing one carboxyl group, one thiol group and a pyridyl N donor atom. Some complexes based on the 2-MN^2-^ ligand have been previously investigated, e.g. by Sun *et al.* (2011). Recently, on the basis of the 2-H_2_MN ligand, we have designed a new multi-carboxylate ligand, 2-carboxymethylsulfanyl nicotinic acid (2-H_2_CMSN) to construct novel complexes (Jiang *et al.*, 2010; 2012). The 2-H_2_CMSN ligand is interesting because of its potential versatile coordination behavior, resulting from one rigid and one flexible carboxyl group. Due to the flexible carboxyl group, it is favorable for constructing novel network structures. Here we report the structure of the new title compound, [Co(2-CMSN)(4,4'-bipy)_1.5_(H_2_O)]^.^0.65H_2_O, (I).

Complex (I) is isostructural to [Ni(2-CMSN)(4,4'-bipy)_1.5_(H_2_O)].0.75H_2_O (Jiang *et al.*, 2012). The asymmetric unit of (I) contains one Co^II^ ion, one 2-CMSN^2-^ ligand, one and a half 4,4-bipy molecules (the other half being completed by inversion symmetry), one coordination water molecule and disordered lattice water molecules with an overall occupancy of 0.65. The coordination environment of the Co^II^ ion is illustrated in Fig. 1. The Co^II^ ion is six-coordinated in a slightly distorted octahedral CoN_3_O_3_ environment: two O atoms originate from one flexible carboxyl group and one rigid carboxyl group of two symmetry-related 2-CMSN^2-^ ligands, three N atoms from three 4,4'-bipy molecules and one O atom from the water molecule. Two adjacent Co^II^ ions are linked by two 2-CMSN^2-^ ligands to give a dinuclear [Co_2_(2-CMSN)_2_] subunit with a Co···Co distance of 6.8600 (3) Å (Fig. 2). The dinuclear [Co_2_(2-CMSN)_2_] subunits are further bridged by 4,4'-bipy linkers to generate a final three-dimensional structure (Fig. 2). The disordered water molecules are situated in the free space of the resulting network. The 4,4'-bipy molecule that is situated on a centre of inversion is exactly planar, whereas the other has a dihedral angle between the two pyridyl rings [N2,C9—C13] and [N3, C14—C18] of 33.16 (7)°.

In the crystal, intra- and inter-subunit O—H···O hydrogen bonds (Table 1) between the coordinating water molecule and carboxylate O atoms enhance the stability of the structure. Although the H atom position of the lattice water molecules could not be located, the O2W···O2 and O3W···S1 contacts of 2.864 (5) Å and 3.724 (9) Å, respectively, suggest also participation of these molecules in hydrogen bonding.

### Experimental

A mixture of 2-H_2_CMSN (0.4 mmol, 0.086 g), CoCl_2_ (0.4 mmol, 0.095 g) and 4,4'-bipy (0.4 mmol, 0.062 g) in CH_3_CH_2_OH (2 ml)/H_2_O (18 ml) was stirred for 1 h. The pH value was adjusted to around 6.0 by sodium carbonate solution in the entire process. Then the mixture was placed in a 25 ml stainless steel reactor and heated at 383 K for 24 h, and then cooled to room temperature for 24 h gave red crystals (yield 46%).

### Refinement

The carbon-bound H-atoms were placed in idealized positions [(C—H = 0.93 or 0.97 Å, *U*_iso_(H) = 1.2Ueq(C)]. The coordinating water H-atoms were located in a different Fourier map and were refined with an O—H distance restrained to 0.85 (2) Å [*U*_iso_(H) = 1.2Ueq(O)]. The two lattice water molecules are occupationally disordered (occupancies of 0.4 for OW2 and 0.25 for OW3). No reasonable H positions could be determined from Fourier maps for these atoms. Therefore the H atoms of OW2 and OW3 were omitted from refinement, but included in the final chemical formula.

### Crystal data

**Table:**

[Co_2_(C_8_H_5_NO_4_S)_2_(C_10_H_8_N_2_)_3_(H_2_O)_2_]·1.3H_2_O	*F*(000) = 1092.9
*M**_r_* = 1068.26	*V*=2340.92(5)Å^3^
Monoclinic, *P*2_1_/*c*	*D*_x_ = 1.516 Mg m^−^^3^
Hall symbol: -P 2ybc	Mo *K*α radiation, λ = 0.71073 Å
*a* = 10.2211 (1) Å	θ = 1.9–27.6°
*b* = 17.1355 (2) Å	µ = 0.87 mm^−^^1^
*c* = 16.4142 (2) Å	*T* = 296 K
β = 125.484 (1)°	Block, red
*V* = 2340.92 (5) Å^3^	0.34 × 0.20 × 0.11 mm
*Z* = 2	

### Data collection

**Table:**

Bruker APEXII CCD diffractometer	5439 independent reflections
Radiation source: fine-focus sealed tube	4772 reflections with *I* > 2σ(*I*)
Graphite monochromator	*R*_int_ = 0.025
ω scans	θ_max_ = 27.6°, θ_min_ = 1.9°
Absorption correction: multi-scan (*SADABS*; Bruker, 2006)	*h* = −13→13
*T*_min_ = 0.814, *T*_max_ = 0.912	*k* = −22→22
38093 measured reflections	*l* = −21→21

### Refinement

**Table:**

Refinement on *F*^2^	Primary atom site location: structure-invariant direct methods
Least-squares matrix: full	Secondary atom site location: difference Fourier map
*R*[*F*^2^ > 2σ(*F*^2^)] = 0.033	Hydrogen site location: inferred from neighbouring sites
*wR*(*F*^2^) = 0.093	H-atom parameters constrained
*S* = 1.05	*w* = 1/[σ^2^(*F*_o_^2^) + (0.0477*P*)^2^ + 1.3453*P*] where *P* = (*F*_o_^2^ + 2*F*_c_^2^)/3
5439 reflections	(Δ/σ)_max_ = 0.001
315 parameters	Δρ_max_ = 0.68 e Å^−^^3^
3 restraints	Δρ_min_ = −0.53 e Å^−^^3^

### Special details

**Table:**

Geometry. All e.s.d.'s (except the e.s.d. in the dihedral angle between two l.s. planes) are estimated using the full covariance matrix. The cell e.s.d.'s are taken into account individually in the estimation of e.s.d.'s in distances, angles and torsion angles; correlations between e.s.d.'s in cell parameters are only used when they are defined by crystal symmetry. An approximate (isotropic) treatment of cell e.s.d.'s is used for estimating e.s.d.'s involving l.s. planes.
Refinement. Refinement of *F*^2^ against ALL reflections. The weighted *R*-factor *wR* and goodness of fit *S* are based on *F*^2^, conventional *R*-factors *R* are based on *F*, with *F* set to zero for negative *F*^2^. The threshold expression of *F*^2^ > σ(*F*^2^) is used only for calculating *R*-factors(gt) *etc*. and is not relevant to the choice of reflections for refinement. *R*-factors based on *F*^2^ are statistically about twice as large as those based on *F*, and *R*- factors based on ALL data will be even larger.

### Fractional atomic coordinates and isotropic or equivalent isotropic displacement parameters (Å^2^)

**Table:**

	*x*	*y*	*z*	*U*_iso_*/*U*_eq_	Occ. (<1)
Co1	0.22673 (3)	−0.467750 (13)	0.253178 (16)	0.02436 (8)	
S1	−0.26808 (6)	−0.54790 (4)	0.03145 (4)	0.04356 (14)	
N1	−0.3385 (2)	−0.68541 (12)	−0.05863 (14)	0.0512 (5)	
N2	0.04982 (18)	−0.38323 (9)	0.15601 (11)	0.0316 (3)	
N3	−0.60092 (18)	−0.12730 (9)	−0.15781 (11)	0.0317 (3)	
N4	0.14149 (18)	−0.47934 (10)	0.34767 (11)	0.0324 (3)	
O1	0.06128 (15)	−0.55683 (8)	0.16874 (10)	0.0366 (3)	
O2	0.20011 (18)	−0.66643 (9)	0.23590 (11)	0.0506 (4)	
O3	−0.54191 (18)	−0.48099 (12)	−0.24881 (12)	0.0595 (5)	
O4	−0.29489 (15)	−0.53055 (8)	−0.15622 (10)	0.0327 (3)	
O1W	0.41017 (15)	−0.55208 (8)	0.34497 (9)	0.0344 (3)	
H1WB	0.3686	−0.5973	0.3268	0.041*	
H1WA	0.4812	−0.5482	0.3336	0.041*	
O2W	0.3056 (6)	−0.8235 (3)	0.2968 (4)	0.0716 (13)*	0.40
O3W	−0.7292 (11)	−0.3553 (6)	−0.2320 (7)	0.082 (2)*	0.25
C1	−0.0421 (3)	−0.75259 (13)	0.07695 (19)	0.0537 (6)	
H1A	0.0564	−0.7762	0.1229	0.064*	
C2	−0.1648 (4)	−0.79492 (14)	−0.0044 (2)	0.0737 (9)	
H2A	−0.1496	−0.8467	−0.0141	0.088*	
C3	−0.3083 (4)	−0.75831 (15)	−0.0694 (2)	0.0661 (8)	
H3A	−0.3895	−0.7863	−0.1245	0.079*	
C4	−0.2204 (2)	−0.64420 (12)	0.01904 (13)	0.0350 (4)	
C5	−0.0664 (2)	−0.67566 (11)	0.08956 (14)	0.0345 (4)	
C6	0.0760 (2)	−0.62946 (11)	0.17223 (13)	0.0314 (4)	
C7	−0.4499 (3)	−0.52819 (15)	−0.09011 (16)	0.0474 (5)	
H7A	−0.5211	−0.5727	−0.1103	0.057*	
H7B	−0.5031	−0.4837	−0.0847	0.057*	
C8	−0.4259 (2)	−0.51158 (12)	−0.17142 (14)	0.0347 (4)	
C9	−0.1027 (3)	−0.26859 (15)	0.12914 (17)	0.0637 (8)	
H9A	−0.1169	−0.2245	0.1563	0.076*	
C10	0.0229 (3)	−0.31873 (14)	0.18932 (16)	0.0563 (7)	
H10A	0.0930	−0.3069	0.2570	0.068*	
C11	−0.0478 (2)	−0.39688 (11)	0.05773 (13)	0.0334 (4)	
H11A	−0.0282	−0.4403	0.0323	0.040*	
C12	−0.1767 (2)	−0.34941 (11)	−0.00793 (13)	0.0346 (4)	
H12A	−0.2421	−0.3614	−0.0758	0.042*	
C13	−0.2083 (2)	−0.28423 (11)	0.02741 (14)	0.0369 (4)	
C14	−0.4239 (3)	−0.19437 (14)	−0.00281 (15)	0.0474 (5)	
H14A	−0.3913	−0.2030	0.0624	0.057*	
C15	−0.3472 (2)	−0.23228 (11)	−0.03857 (14)	0.0359 (4)	
C16	−0.5485 (2)	−0.14378 (13)	−0.06392 (14)	0.0421 (5)	
H16A	−0.5990	−0.1197	−0.0383	0.051*	
C17	−0.5296 (3)	−0.16573 (12)	−0.19305 (15)	0.0422 (5)	
H17A	−0.5660	−0.1568	−0.2590	0.051*	
C18	−0.4046 (3)	−0.21800 (12)	−0.13694 (15)	0.0439 (5)	
H18A	−0.3595	−0.2434	−0.1652	0.053*	
C19	−0.0124 (3)	−0.46731 (18)	0.31159 (16)	0.0580 (7)	
H19A	−0.0828	−0.4523	0.2450	0.070*	
C20	−0.0725 (2)	−0.47589 (19)	0.36765 (16)	0.0623 (8)	
H20A	−0.1810	−0.4673	0.3384	0.075*	
C21	0.1870 (2)	−0.51257 (11)	0.50404 (14)	0.0342 (4)	
H21A	0.2596	−0.5290	0.5698	0.041*	
C22	0.2363 (2)	−0.50319 (11)	0.44222 (14)	0.0334 (4)	
H22A	0.3429	−0.5143	0.4684	0.040*	
C23	0.0286 (2)	−0.49728 (11)	0.46761 (13)	0.0319 (4)	

### Atomic displacement parameters (Å^2^)

**Table:**

	*U*^11^	*U*^22^	*U*^33^	*U*^12^	*U*^13^	*U*^23^
Co1	0.01954 (12)	0.02930 (13)	0.02172 (12)	0.00108 (8)	0.01053 (10)	0.00026 (8)
S1	0.0376 (3)	0.0610 (3)	0.0279 (2)	0.0119 (2)	0.0166 (2)	−0.0005 (2)
N1	0.0453 (10)	0.0565 (11)	0.0372 (9)	−0.0174 (9)	0.0156 (8)	−0.0018 (8)
N2	0.0287 (7)	0.0332 (7)	0.0277 (7)	0.0085 (6)	0.0133 (6)	0.0012 (6)
N3	0.0272 (7)	0.0340 (8)	0.0269 (7)	0.0054 (6)	0.0118 (6)	0.0025 (6)
N4	0.0243 (7)	0.0467 (9)	0.0270 (7)	−0.0038 (6)	0.0153 (6)	−0.0027 (6)
O1	0.0280 (7)	0.0316 (6)	0.0355 (7)	−0.0012 (5)	0.0100 (6)	−0.0022 (5)
O2	0.0419 (8)	0.0390 (8)	0.0445 (8)	0.0039 (6)	0.0101 (7)	0.0102 (6)
O3	0.0285 (8)	0.1104 (15)	0.0385 (8)	0.0196 (8)	0.0188 (7)	0.0218 (9)
O4	0.0257 (6)	0.0449 (7)	0.0285 (6)	0.0045 (5)	0.0163 (5)	−0.0001 (5)
O1W	0.0265 (6)	0.0432 (7)	0.0298 (6)	0.0052 (5)	0.0142 (5)	0.0048 (5)
C1	0.0587 (14)	0.0325 (10)	0.0540 (13)	−0.0024 (10)	0.0237 (12)	0.0045 (9)
C2	0.095 (2)	0.0312 (11)	0.0711 (18)	−0.0167 (13)	0.0345 (17)	−0.0085 (11)
C3	0.0711 (18)	0.0491 (14)	0.0481 (13)	−0.0264 (13)	0.0174 (13)	−0.0067 (11)
C4	0.0346 (10)	0.0435 (10)	0.0276 (8)	−0.0089 (8)	0.0184 (8)	0.0004 (7)
C5	0.0379 (10)	0.0326 (9)	0.0317 (9)	−0.0069 (7)	0.0196 (8)	0.0025 (7)
C6	0.0324 (9)	0.0345 (9)	0.0262 (8)	−0.0014 (7)	0.0164 (7)	0.0043 (7)
C7	0.0287 (10)	0.0808 (16)	0.0351 (10)	0.0118 (10)	0.0198 (9)	0.0087 (10)
C8	0.0239 (9)	0.0502 (11)	0.0280 (8)	−0.0007 (8)	0.0140 (7)	−0.0029 (8)
C9	0.0586 (15)	0.0597 (14)	0.0353 (11)	0.0329 (12)	0.0057 (10)	−0.0140 (10)
C10	0.0495 (13)	0.0581 (14)	0.0293 (10)	0.0250 (11)	0.0046 (9)	−0.0102 (9)
C11	0.0403 (10)	0.0324 (9)	0.0287 (8)	0.0094 (7)	0.0207 (8)	0.0026 (7)
C12	0.0402 (10)	0.0353 (9)	0.0240 (8)	0.0088 (8)	0.0162 (8)	0.0023 (7)
C13	0.0360 (10)	0.0374 (10)	0.0292 (9)	0.0131 (8)	0.0143 (8)	0.0026 (7)
C14	0.0505 (12)	0.0582 (13)	0.0276 (9)	0.0263 (10)	0.0193 (9)	0.0095 (9)
C15	0.0335 (10)	0.0350 (9)	0.0294 (9)	0.0100 (7)	0.0127 (8)	0.0010 (7)
C16	0.0421 (11)	0.0503 (11)	0.0334 (10)	0.0189 (9)	0.0216 (9)	0.0063 (8)
C17	0.0502 (12)	0.0444 (11)	0.0301 (9)	0.0165 (9)	0.0222 (9)	0.0083 (8)
C18	0.0508 (12)	0.0454 (11)	0.0371 (10)	0.0202 (9)	0.0265 (10)	0.0073 (9)
C19	0.0243 (10)	0.123 (2)	0.0231 (9)	−0.0001 (11)	0.0117 (8)	0.0058 (11)
C20	0.0190 (9)	0.137 (3)	0.0276 (10)	−0.0005 (12)	0.0119 (8)	0.0040 (12)
C21	0.0314 (9)	0.0408 (10)	0.0322 (9)	0.0058 (7)	0.0195 (8)	0.0088 (7)
C22	0.0272 (9)	0.0401 (10)	0.0358 (9)	0.0055 (7)	0.0200 (8)	0.0067 (8)
C23	0.0258 (9)	0.0428 (10)	0.0286 (8)	−0.0074 (7)	0.0166 (7)	−0.0042 (7)

### Geometric parameters (Å, º)

**Table:**

Co1—O4^i^	2.0752 (13)	C5—C6	1.514 (3)
Co1—O1	2.0951 (13)	C7—C8	1.518 (3)
Co1—N2	2.1361 (14)	C7—H7A	0.9700
Co1—O1W	2.1434 (13)	C7—H7B	0.9700
Co1—N4	2.1847 (15)	C9—C10	1.375 (3)
Co1—N3^ii^	2.2141 (15)	C9—C13	1.390 (3)
S1—C4	1.765 (2)	C9—H9A	0.9300
S1—C7	1.801 (2)	C10—H10A	0.9300
N1—C3	1.323 (4)	C11—C12	1.382 (2)
N1—C4	1.340 (3)	C11—H11A	0.9300
N2—C10	1.331 (2)	C12—C13	1.380 (3)
N2—C11	1.336 (2)	C12—H12A	0.9300
N3—C16	1.335 (2)	C13—C15	1.482 (3)
N3—C17	1.338 (2)	C14—C16	1.377 (3)
N3—Co1^iii^	2.2141 (14)	C14—C15	1.384 (3)
N4—C22	1.330 (2)	C14—H14A	0.9300
N4—C19	1.336 (3)	C15—C18	1.382 (3)
O1—C6	1.251 (2)	C16—H16A	0.9300
O2—C6	1.251 (2)	C17—C18	1.384 (3)
O3—C8	1.243 (2)	C17—H17A	0.9300
O4—C8	1.254 (2)	C18—H18A	0.9300
O4—Co1^i^	2.0752 (13)	C19—C20	1.379 (3)
O1W—H1WB	0.8500	C19—H19A	0.9300
O1W—H1WA	0.8500	C20—C23	1.388 (3)
C1—C5	1.379 (3)	C20—H20A	0.9300
C1—C2	1.392 (4)	C21—C22	1.379 (2)
C1—H1A	0.9300	C21—C23	1.387 (2)
C2—C3	1.366 (4)	C21—H21A	0.9300
C2—H2A	0.9300	C22—H22A	0.9300
C3—H3A	0.9300	C23—C23^iv^	1.484 (3)
C4—C5	1.412 (3)		
			
O4^i^—Co1—O1	89.07 (5)	C8—C7—H7B	108.6
O4^i^—Co1—N2	87.27 (5)	S1—C7—H7B	108.6
O1—Co1—N2	89.52 (6)	H7A—C7—H7B	107.5
O4^i^—Co1—O1W	89.06 (5)	O3—C8—O4	125.96 (18)
O1—Co1—O1W	90.84 (5)	O3—C8—C7	115.63 (17)
N2—Co1—O1W	176.31 (5)	O4—C8—C7	118.40 (17)
O4^i^—Co1—N4	173.20 (6)	C10—C9—C13	119.52 (19)
O1—Co1—N4	84.38 (6)	C10—C9—H9A	120.2
N2—Co1—N4	94.48 (6)	C13—C9—H9A	120.2
O1W—Co1—N4	89.21 (5)	N2—C10—C9	123.60 (19)
O4^i^—Co1—N3^ii^	91.36 (5)	N2—C10—H10A	118.2
O1—Co1—N3^ii^	179.27 (6)	C9—C10—H10A	118.2
N2—Co1—N3^ii^	89.91 (6)	N2—C11—C12	122.98 (16)
O1W—Co1—N3^ii^	89.76 (6)	N2—C11—H11A	118.5
N4—Co1—N3^ii^	95.21 (6)	C12—C11—H11A	118.5
C4—S1—C7	102.94 (11)	C13—C12—C11	119.87 (17)
C3—N1—C4	118.3 (2)	C13—C12—H12A	120.1
C10—N2—C11	117.00 (16)	C11—C12—H12A	120.1
C10—N2—Co1	122.93 (13)	C12—C13—C9	116.92 (17)
C11—N2—Co1	119.88 (12)	C12—C13—C15	122.51 (17)
C16—N3—C17	116.14 (16)	C9—C13—C15	120.57 (17)
C16—N3—Co1^iii^	123.35 (12)	C16—C14—C15	119.91 (18)
C17—N3—Co1^iii^	120.09 (12)	C16—C14—H14A	120.0
C22—N4—C19	115.95 (16)	C15—C14—H14A	120.0
C22—N4—Co1	122.38 (12)	C18—C15—C14	116.83 (17)
C19—N4—Co1	121.55 (13)	C18—C15—C13	122.48 (18)
C6—O1—Co1	131.70 (12)	C14—C15—C13	120.68 (18)
C8—O4—Co1^i^	130.24 (12)	N3—C16—C14	123.75 (18)
Co1—O1W—H1WB	108.2	N3—C16—H16A	118.1
Co1—O1W—H1WA	107.6	C14—C16—H16A	118.1
H1WB—O1W—H1WA	108.2	N3—C17—C18	123.70 (18)
C5—C1—C2	120.1 (2)	N3—C17—H17A	118.1
C5—C1—H1A	119.9	C18—C17—H17A	118.1
C2—C1—H1A	119.9	C15—C18—C17	119.59 (18)
C3—C2—C1	118.1 (2)	C15—C18—H18A	120.2
C3—C2—H2A	121.0	C17—C18—H18A	120.2
C1—C2—H2A	121.0	N4—C19—C20	123.60 (19)
N1—C3—C2	123.9 (2)	N4—C19—H19A	118.2
N1—C3—H3A	118.1	C20—C19—H19A	118.2
C2—C3—H3A	118.1	C19—C20—C23	120.19 (19)
N1—C4—C5	122.6 (2)	C19—C20—H20A	119.9
N1—C4—S1	116.49 (16)	C23—C20—H20A	119.9
C5—C4—S1	120.88 (14)	C22—C21—C23	119.62 (17)
C1—C5—C4	117.00 (19)	C22—C21—H21A	120.2
C1—C5—C6	118.12 (19)	C23—C21—H21A	120.2
C4—C5—C6	124.70 (17)	N4—C22—C21	124.35 (17)
O2—C6—O1	125.36 (17)	N4—C22—H22A	117.8
O2—C6—C5	117.72 (17)	C21—C22—H22A	117.8
O1—C6—C5	116.86 (16)	C21—C23—C20	116.18 (17)
C8—C7—S1	114.81 (14)	C21—C23—C23^iv^	121.7 (2)
C8—C7—H7A	108.6	C20—C23—C23^iv^	122.1 (2)
S1—C7—H7A	108.6		
			
O4^i^—Co1—N2—C10	−135.1 (2)	C1—C5—C6—O1	164.59 (19)
O1—Co1—N2—C10	135.8 (2)	C4—C5—C6—O1	−10.3 (3)
N4—Co1—N2—C10	51.5 (2)	C4—S1—C7—C8	−75.84 (19)
N3^ii^—Co1—N2—C10	−43.7 (2)	Co1^i^—O4—C8—O3	−8.2 (3)
O4^i^—Co1—N2—C11	49.97 (15)	Co1^i^—O4—C8—C7	170.55 (14)
O1—Co1—N2—C11	−39.12 (15)	S1—C7—C8—O3	−164.40 (18)
O1W—Co1—N2—C11	56.5 (9)	S1—C7—C8—O4	16.8 (3)
N4—Co1—N2—C11	−123.44 (15)	C11—N2—C10—C9	3.2 (4)
N3^ii^—Co1—N2—C11	141.34 (15)	Co1—N2—C10—C9	−171.9 (2)
O4^i^—Co1—N4—C22	99.9 (4)	C13—C9—C10—N2	−0.7 (5)
O1—Co1—N4—C22	115.53 (15)	C10—N2—C11—C12	−3.0 (3)
N2—Co1—N4—C22	−155.40 (15)	Co1—N2—C11—C12	172.19 (15)
O1W—Co1—N4—C22	24.61 (15)	N2—C11—C12—C13	0.5 (3)
N3^ii^—Co1—N4—C22	−65.08 (16)	C11—C12—C13—C9	2.0 (3)
O4^i^—Co1—N4—C19	−76.0 (5)	C11—C12—C13—C15	−178.22 (19)
O1—Co1—N4—C19	−60.33 (19)	C10—C9—C13—C12	−1.9 (4)
N2—Co1—N4—C19	28.75 (19)	C10—C9—C13—C15	178.3 (3)
O1W—Co1—N4—C19	−151.25 (19)	C16—C14—C15—C18	−1.5 (3)
N3^ii^—Co1—N4—C19	119.06 (19)	C16—C14—C15—C13	177.3 (2)
O4^i^—Co1—O1—C6	85.94 (17)	C12—C13—C15—C18	−33.7 (3)
N2—Co1—O1—C6	173.22 (17)	C9—C13—C15—C18	146.1 (3)
O1W—Co1—O1—C6	−3.11 (17)	C12—C13—C15—C14	147.5 (2)
N4—Co1—O1—C6	−92.23 (17)	C9—C13—C15—C14	−32.7 (3)
N3^ii^—Co1—O1—C6	−148 (4)	C17—N3—C16—C14	2.8 (3)
C5—C1—C2—C3	−0.8 (4)	Co1^iii^—N3—C16—C14	−169.71 (18)
C4—N1—C3—C2	1.9 (4)	C15—C14—C16—N3	−1.0 (4)
C1—C2—C3—N1	−1.3 (5)	C16—N3—C17—C18	−2.2 (3)
C3—N1—C4—C5	−0.4 (3)	Co1^iii^—N3—C17—C18	170.62 (18)
C3—N1—C4—S1	−178.79 (19)	C14—C15—C18—C17	2.1 (3)
C7—S1—C4—N1	−16.68 (17)	C13—C15—C18—C17	−176.7 (2)
C7—S1—C4—C5	164.86 (15)	N3—C17—C18—C15	−0.3 (4)
C2—C1—C5—C4	2.1 (3)	C22—N4—C19—C20	2.2 (4)
C2—C1—C5—C6	−173.2 (2)	Co1—N4—C19—C20	178.3 (2)
N1—C4—C5—C1	−1.6 (3)	N4—C19—C20—C23	0.8 (5)
S1—C4—C5—C1	176.78 (16)	C19—N4—C22—C21	−2.8 (3)
N1—C4—C5—C6	173.38 (18)	Co1—N4—C22—C21	−178.90 (15)
S1—C4—C5—C6	−8.3 (3)	C23—C21—C22—N4	0.6 (3)
Co1—O1—C6—O2	4.9 (3)	C22—C21—C23—C20	2.4 (3)
Co1—O1—C6—C5	−172.18 (12)	C22—C21—C23—C23^iv^	−177.0 (2)
C1—C5—C6—O2	−12.7 (3)	C19—C20—C23—C21	−3.0 (4)
C4—C5—C6—O2	172.36 (18)	C19—C20—C23—C23^iv^	176.3 (3)

Symmetry codes: (i) −*x*, −*y*−1, −*z*; (ii) *x*+1, −*y*−1/2, *z*+1/2; (iii) *x*−1, −*y*−1/2, *z*−1/2; (iv) −*x*, −*y*−1, −*z*+1.

### Hydrogen-bond geometry (Å, º)

**Table:**

*D*—H···*A*	*D*—H	H···*A*	*D*···*A*	*D*—H···*A*
O1*W*—H1*WA*···O3^i^	0.85	1.89	2.663 (2)	150
O1*W*—H1*WB*···O2	0.85	1.90	2.682 (2)	152

Symmetry code: (i) −*x*, −*y*−1, −*z*.

## References

[bb1] Brandenburg, K. (2008). *DIAMOND* Crystal Impact GbR, Bonn, Germany.

[bb2] Bruker (2006). *APEX2*, *SAINT* and *SADABS* Bruker AXS Inc., Madison, Wisconsin, USA.

[bb3] Jiang, X.-R., Wang, X.-J. & Feng, Y.-L. (2010). *Acta Cryst.* E**66**, o3308. 10.1107/S1600536810048385 PMC3011421 21589586

[bb4] Jiang, X.-R., Wang, X.-J. & Feng, Y.-L. (2012). *Inorg. Chim. Acta*, **383**, 38–45.

[bb5] Sheldrick, G. M. (2008). *Acta Cryst.* A**64**, 112–122. 10.1107/S0108767307043930 18156677

[bb6] Sun, D., Wang, D.-F., Han, X.-G., Zhang, N., Huang, R.-B. & Zheng, L.-S. (2011). *Chem. Commun.* **47**, 746–748. 10.1039/c0cc03534c 21072394

[bb7] Wang, X.-L., Qin, C., Wang, E.-B., Xu, L., Su, Z.-M. & Hu, C.-W. (2004). *Angew. Chem. Int. Ed.* **43**, 5036–5040. 10.1002/anie.200460758 15384113

